# The role of a team psychological safety feeling in teamwork in the classroom

**DOI:** 10.1016/j.heliyon.2024.e37618

**Published:** 2024-09-07

**Authors:** Dalibor Gonda, Anna Tirpáková, Gabriela Pavlovičová, Viliam Ďuriš

**Affiliations:** a Department of Mathematical Methods and Operations Research, Faculty of Management Science and Informatics, University of Žilina, Univerzitná 1, 01026, Žilina, Slovakia; b Department of Mathematics, Faculty of Natural Sciences and Informatics, Constantine the Philosopher University in Nitra, Tr. A. Hlinku 1, 94901, Nitra, Slovakia; c Department of School Education, Faculty of Humanities, Tomas Bata University in Zlin, Stefanikova 5670, 76001, Zlin, Czech Republic

**Keywords:** Active cooperation, Feeling of psychological safety, Detecting mistakes, Learning from mistakes, Active learning

## Abstract

Active cooperation is expected from the student in the education center, which is associated with fears of expressing their opinions, because a possible mistake may result in a negative reaction from the environment. In our research, we investigated the impact of team psychological safety feelings on students' willingness to engage in active learning. 244 students aged 18 and 19 participated in the research. A mixed methods approach was used to obtain the necessary data. By data analysis, we revealed three separate dimensions in respondents' answers to questionnaire items. According to our findings, students' involvement in active learning is strongly supported by their internal motivation to acquire new knowledge and trust in the class collective. A sense of psychological team safety has an important place in encouraging the student to engage in common activities. At the same time, we found that the teacher has a decisive influence on building the student's trust in the class team.

## 1 Introduction

Currently, in education, great emphasis is placed on the student, who should be the center of the educational process [1]. One of the important skills that employers expect from school graduates is teamwork. Therefore, forms of active learning are preferred as part of teaching, and the student is expected to be actively and creatively involved in teamwork [2]. Teamwork requires social interaction from individual team members in the form of cognitive, verbal and behavioral activities aimed at achieving a collective goal [3,4]. In the school environment, teamwork is applied in the form of cooperative teaching, in which social interactions of students are combined with educational activities [5]. Cooperative learning is learning in small groups based on the theory of social interdependence [6]. Its essence is the cooperation of students in order to maximize their learning and each other's learning [7]. In preparation for cooperative learning, the teacher sets goals, plans and structures tasks, and assigns subtasks to students to work together on a common solution [8,9]. In the lesson, the teacher assumes the role of facilitator and directs the students' work [10]. Cooperative teaching is a structured form of learning that requires the active participation of each student and its critical element is the teamwork of all group members [11]. If team members cannot successfully share their knowledge, coordinate behavior and trust each other, i.e. work as a team, the team fails to solve the task despite extensive knowledge of the assigned task [12]. One of the frequent causes of insufficient cooperation in a group is the fact that participation or expected participation in a social activity may cause fear of negative evaluation [13]. Participating or anticipating participation in a social activity can cause fear of negative evaluation [13]. Students may fear that their ideas will be negatively evaluated by classmates or the teacher [14]. According to Conlin & Scherr [15], the form of active learning further increases students' fear of negative evaluation and increases their fear of making a mistake.

In general, error refers to unintentional deviation from goals, rules, and norms [16]. Making mistakes is a part of every creative process and everyday life, so they also have their place in preparing students for their professional life. One of the key tasks of the teacher is to create such a classroom climate where students are encouraged to express their opinions without fear of negative evaluation [17], i.e., an environment in which students perceive that their surroundings value them little or not at all [18]. According to Tulis [19], it is the behavior of teachers towards students' mistakes that has a major impact on students' attitudes towards their own mistakes and learning from mistakes. In general, teachers approach students' errors adaptively or maladaptively. An adaptive approach consists, for example, in a discussion with the whole class, emphasizing the learning potential of mistakes and preventing negative reactions from classmates. In contrast, a maladaptive approach is manifested by criticizing a student for making a mistake and asking another student to correct a classmate's mistake [19,20]. With an adaptive approach to mistakes, teachers should create a positive error climate in teaching and encourage students to learn from mistakes [21].

Error framing is used as a pedagogical tool to reduce students' fears [22]. Error framing involves framing mistakes or misconceptions as natural and useful [23]. Error framing can, in addition to reducing students' fear of making mistakes, increase students' motivation to express their suggestions for problem solutions [24]. Another task of the teacher in creating a positive error climate in the classroom is to achieve a change in the students' attitude towards their own mistakes, which they made while solving the task. Research has shown that students are not only afraid of making a mistake [25], but they try to hide their mistakes [26]. In order for an individual to decide to reveal a mistake to others, a sense of team psychological safety is needed. Team psychological safety is defined as the shared belief that the team is safe from interpersonal risks [27]. This construct represents a sense of trust that the team will not shame, punish, or reject anyone for speaking up. An atmosphere of team psychological safety alleviates fears about the reactions of others and thus facilitates the individual's involvement in common work [28]. According to Boaler [29], a mistake made by a student is very useful because it is an opportunity for learning. If the student perceives his own mistake in this way and has a sense of team psychological safety, he will be more motivated to reveal his own mistakes. In order for the learning benefit of revealing one's own mistakes to be as high as possible, it is necessary to provide the student with corrective feedback [30]. Research points to the fact that students do not need to be aware of the positive effect of mistakes in order to benefit from them in learning, but this lack of awareness limits the benefit of mistakes in their progress [31]. According to Metcalfe and Xu [32], own mistakes are more beneficial than others' mistakes for the correct acquisition of new knowledge. In order for learning from one's own mistakes to take place, it is necessary for learning to take place in such a social environment that supports students in revealing their own mistakes. In the available literature, a positive classroom error climate is assumed to be such a supportive environment, but the impact of team psychological safety in the school environment is less explored [33]. The aim of our research was to find out the influence of the feeling of team psychological safety on students' decision whether to reveal the mistake they have committed in front of the class group.

## 2 Theoretical basis

Teaching at all stages of the educational process is moving to a form of active learning because active learning is a more effective way of teaching [34]. Active learning is usually understood as the common work of students during classes, associated with more frequent evaluation by the teacher and the classmates [22]. Some research studies revealed that students experienced fear of making a mistake and being negatively evaluated during active learning ([14,35]). In the following text, we will understand a mistake as a mistake and not a slip. Slips, in contrast to mistakes, are not the result of incorrect learning of the subject matter or incorrect reasoning, but the result of chance or inattention [36]. When students make mistakes, they view them as personal failures, further limiting their participation in active learning [37]. Errors must be clearly distinguished from failure, which is a combination of errors, rule violations, and random factors [38]. Error itself may not lead to failure because it can be detected and corrected immediately, or it can occur in a safe environment where negative evaluation does not occur [39]. According to Mangels et al. [40] in the school environment, a mistake is perceived as an indicator of poor performance, insufficient preparation of students and sometimes also as a lack of intelligence. For this reason, it is natural that students try to avoid making mistakes or try to hide them in order to prevent a decrease in their own value in the eyes of the teacher and classmates [41].

A general aversion to making mistakes during teaching and learning has been identified in students. At the same time, students showed an effort to learn from mistakes when they have already made them [42]. According to Reason [43], the human cognitive apparatus, created for heuristic processing, is prone to errors and not for error-free algorithmic processing, therefore it is not completely possible to prevent errors. A heuristic approach to problem solving is natural for humans, which is associated with a certain fumbling, from which errors can originate. In this context, it is very important that students learn to positively perceive and manage mistakes, as these elements have a positive impact on learning [[44], [45], [46]]. According to Tulis [19], teachers' attitude towards their own mistakes and students' mistakes has a major influence on students' perception of mistakes. By normalizing incorrect answers, teachers contribute to reducing the fear of negative evaluation. With this approach, they create an awareness of naturalness in students, thereby encouraging students to engage in active learning [23].

Professional literature mentions error framing as an effective tool for reducing students' fears about making mistakes. The teacher can apply error framing in the classroom, for example, by explicitly expressing the opinion in the classroom that it is completely natural to answer the questions asked incorrectly, or by marking the wrong answer as a common mistake that students make when solving this type of task [14]. In this way, the teacher expresses his positive attitude towards mistakes, which will gradually be transferred to the students and, according to Steele-Johnson & Kalinoski [24], will also increase the motivation of students to engage in active learning in the classroom. A teacher's positive attitude towards mistakes is enhanced by his positive reactions to students' mistakes. It is the teacher's positive reaction to mistakes that has a fundamental impact on students perceiving a mistake not only as a natural element of learning, but also as an opportunity for learning ([19,47]). According to Piaget [48], learning begins with the balance of the mind and the student tries to incorporate new knowledge into his world of thought. If the new knowledge cannot be integrated into his mental models, an imbalance occurs, which requires changing the given mental model or correcting the perception of the new knowledge. In both cases, we can talk about the need to eliminate the mistake the student made. After its removal, everything fits together again, and a state of balance occurs again in the mind [48].

From the perspective of adaptive neurocognitive theory, errors cause a cognitive discrepancy that activates conflict resolution processes [49]. According to Butler et al. [50], errors causing an imbalance (discrepancy) of the mind are unlikely to be corrected spontaneously. Therefore, the teacher's intervention is necessary in the form of corrective feedback that the student receives after making a mistake [31]. This will not only correct the mistake and restore balance in the mind, but also facilitate learning ([36,51]). If a student makes a mistake while solving a problem, it is not enough to mark his solution as faulty. The feedback he receives has the character of corrective feedback only if it contains a clearly formulated correct answer [52], on the basis of which the student learns where he made a mistake. Subsequently, the teacher can analyze the cause of the mistake made with the student. There is research that shows that learning from mistakes is most effective when students learn from their own mistakes rather than from mistakes made by someone else ([32,53]). The effect of learning from errors can be enhanced if the student receives corrective feedback shortly after making the error (e.g. Refs. [[53], [54], [55]]).

Immediate corrective feedback prevents learning of incorrect responses and allows for re-engagement in problem solving [56]. It follows from the overview that the teacher can normalize mistakes with his attitude and use students' mistakes for learning through timely corrective feedback. In order for learning from mistakes to be possible and as effective as possible, it is necessary for students to be willing to reveal the mistakes they have made. Therefore, it is important that learning takes place in an environment that minimizes students' fear of failure, thereby opening up space for learning from mistakes. To describe the degree to which the environment supports learning from mistakes in school education, experts have introduced the term "error climate" or "error culture" (e.g. Refs. [57,58]). In the following text, we will prefer the term "error climate", which includes the quality and quantity of verbal and non-verbal interactions within the classroom [59]. It follows from the above text that the error climate is primarily determined by the teacher's attitude towards errors. But the reactions of classmates also play a significant role. Error climate is considered a multidimensional construct [60] containing eight subdimensions that can be categorized into three groups [61].

The first group of sub-dimensions relates directly to the teacher and includes: (A) tolerance towards mistakes – includes the potential attitude of tolerance or intolerance of the teacher towards the mistakes made by the student. The attitude of intolerance towards mistakes will manifest itself e.g., verbal statements during class that mistakes should be avoided, or asking questions mostly to students for which he assumes the correct answer. (B) Irrelevance of errors to assessment – refers to the impact of students' errors on their poor assessment. In particular, the interweaving of learning and assessment has an adverse effect on students' motivation to learn and promotes the creation of a negative error climate [62]. (C) Teacher Support for Subsequent Errors - includes assistance provided by a teacher to a student who has made an error. In addition to the necessary help in removing the error caused, an important aspect of the help is the patience of the teacher [57]. (D) Absence of negative reactions from the teacher - includes expressions of anger, annoyance or ridicule of the student who made the mistake. The second group of sub-dimensions of the error climate, which refers to the reactions of classmates, includes: (E) Absence of negative reactions of classmates - includes negative reactions of classmates that cause negative emotions in the one who committed the error, which in turn leads to avoiding or hiding mistakes. In both cases, learning is slowed down. (F) Take the risk of error - describes the nature of the climate that encourages or does not encourage students to take the risk of making an error. The third group of sub-dimensions focuses on the social processes of learning in the classroom and includes: (G) Error analysis - involves open-ended problem solving in a team that promotes learning from mistakes. (H) Functionality of learning errors - refers to the extent to which errors are the starting point of the learning process in the classroom. Based on the mentioned sub-dimensions, the error climate can be conceptualized as hierarchically structured, from which it is clear that several prerequisites must be met before errors can initiate learning [61].

First of all, it is necessary to create a positive error climate in the classroom, in which errors are evaluated and used as an integral element of learning [63]. We consider subdimensions (D) and (E) to be its basis, which are the assumption that the student assumes the risk of making a mistake (subdimension (F)). Only through the subsequent analysis of errors (subdimension (G)) does learning occur, and thus the error made and discovered becomes the starting point for learning in the classroom (subdimension (H)). In order for the student to take the risk of making a mistake, it is necessary for the student to have a sense of team psychological security. Research shows that a positive classroom error climate and students' sense of team psychological safety significantly increase their engagement in learning activities [64]. According to Edmondson [27], team psychological safety is the shared belief that the team is safe from interpersonal risks. This term represents a sense of trust that other team members will not reject, shame, or punish someone for speaking up. This creates a learning environment that helps students express their own ideas and evaluate the new ideas of others [65]. Team psychological safety describes a climate characterized by interpersonal trust and mutual respect in which people can be themselves [27]. These two aspects facilitate learning behavior in the classroom because they mitigate concerns about negative team reactions ([28,66]).

The feeling of team psychological safety motivates individuals to reveal their own mistakes, which subsequently has a positive effect on their educational results [67]. In such a safe environment, the individual feels accepted and can without fear contribute to the solution of the problem by presenting their own thoughts or ideas [68]. Students then enter into communication with the teacher and classmates without fear because the feeling of team psychological safety guarantees acceptance of their individuality and way of expressing themselves. In such a case, students are actively involved in learning within the classroom, they reveal their own mistakes, and thus the feeling of team psychological safety positively affects their learning [69]. For effective learning from mistakes, students' willingness to reveal their own mistakes is crucial. This willingness is supported by the class collective, in which they feel accepted and thus motivated to solve assigned problems together within the learning process. From the review, it follows that the feeling of team psychological safety has the potential to be a motivating factor in the involvement of students in teamwork in the classroom.

## 3 Methodology

Pedagogical research was carried out in selected secondary schools in Slovakia, with the consent of the school management. The aim of the research was to determine the influence of the feeling of team psychological safety on the motivation of students to engage in teamwork within the teaching. The research was conducted at high schools, where various forms of active learning were used. The schools were selected according to this criterion, so that the students have experience in presenting their own ideas and solutions in the class group. We assumed that this fact could contribute to the relevance of the answers obtained from the respondents. In the final selection of high schools, we used Stratified sampling in order to increase the variability of the monitored statistical features [70]. We divided the gymnasiums that met the initial criteria and their principals gave preliminary consent to the research into two groups. In one group there were gymnasiums located in larger cities (12) and in the other group there were gymnasiums located in smaller cities (9). In both lists, the gymnasiums were arranged in alphabetical order, according to the cities in which they are located. Two gymnasiums were selected from each group by simple random sampling (using randomnumbergenerator.org). With the consent of the principals of the four gymnasiums all fourth-year students were approached through class teachers. A total of 285 students from the fourth graders approached came to the meeting with the researchers. At the meetings with the students at individual schools, the students were familiarized with the form and content of pedagogical research. During these meetings, the researchers were careful not to influence their decision to participate in the research or the responses of future respondents when describing the research and responding to the students' questions. After familiarizing themselves with the content and form of the research, 244 students aged 18–19 voluntarily participated in the research itself. All research participants were assured of the anonymous nature of the research.

A mixed methods approach was chosen as the research method, which integrates a quantitative questionnaire method with a qualitative interview [71]. Questionnaire quantitative research makes it possible to obtain the opinions or attitudes of a larger number of respondents. The weaknesses of the questionnaire method are possible shallower answers, or the problem of respondents reliably answering some of the questionnaire items. Therefore, it is advisable to supplement the questionnaire method with an interview, which allows obtaining deeper answers, but from a smaller number of respondents [72]. Based on the above, we supplemented the quantitative questionnaire research with a semi-structured interview with some of the respondents in order to obtain more in-depth answers.

In the quantitative part of the research, a questionnaire was used, the basis of which was the questionnaire of the authors Fischer et al. [73]. We modified the original questionnaire for the school environment. Based on the research carried out by Wilson [2], we also added several other items to the questionnaire, which are related to the motivation of students to engage in active learning (Appendix A). Questionnaire items, i.e., questions Q1-Q16, are compiled in such a way that it is possible to identify the degree of influence of individual variables (Q1-Q16) on the motivation of students to engage in the common solution of tasks within the teaching, and thus also to reveal possible own mistakes. Respondents answered each item by selecting an answer from a seven-point Likert scale: (*weak motivation – 1; very strong motivation – 7).* Interviews were conducted with eight students after the analysis of the quantitative part of the research was completed. The prepared questions used in the interview were designed to follow the findings from the questionnaire part of the research. The aim of the interview was to gain a deeper insight into the structure of motivation to gain new knowledge, which was identified in the quantitative part, as a factor with a strong influence on students' willingness to engage in common activities. All participants consented to the audio recording of the interview, which lasted an average of 30 min. We used a constant comparative method, an inductive coding process [74], to obtain data from individual interviews.

To start the experiment, we stated the following research hypothesis.

H The feeling of team psychological safety is a motivating factor for students' involvement in active learning.

For the analysis of the research results, we used selected statistical methods, namely methods of descriptive statistics, the correlation coefficient between the observed characteristics and factor analysis, which we will briefly describe in parallel with the data analysis.

## 4 Data analysis

Before the actual statistical analysis of the data obtained by the questionnaire method, the validity and reliability of the data was first verified. Given that there is a mutual relationship between reliability and validity (good reliability is a necessary condition for correct validity), in our case we calculated reliability, to verify the reliability of the data. Reliability, or the accuracy of the data, describes the impact of random errors on the result of the selected statistical test and how reproducible the result is. If we want our chosen statistical method to measure real skills as accurately as possible, we must suppress the influence of chance as much as possible, i.e. determine reliability as a measure of dependence between the individual items of the obtained data. Cronbach's alpha is used to unambiguously determine the reliability (internal consistency of the test) (e.g. Ref. [75]). Its value is given by the relation:

$$\alpha =\frac{k}{k-1}\left(1-\frac{\sum_{j=1}^{k}var\left(Y_{j}\right)}{var\left(Y\right)}\right)$$

where k is the number of items in the test, var(Y_j_) is the variance of the points of the item j, and var(Y) is the variance of the raw test scores ([76,77]). Currently, the calculation of Cronbach's alpha is part of statistical software.

In our case, the value of Cronbach's alpha α = 0.843 was calculated using the STATISTICA program. This value points to a very strong linear dependence of the questionnaire items (the influence of random errors on the test result is very small), i.e.the value α = 0.843 confirms the reliability of the obtained data [76].

**Fig. 1:**
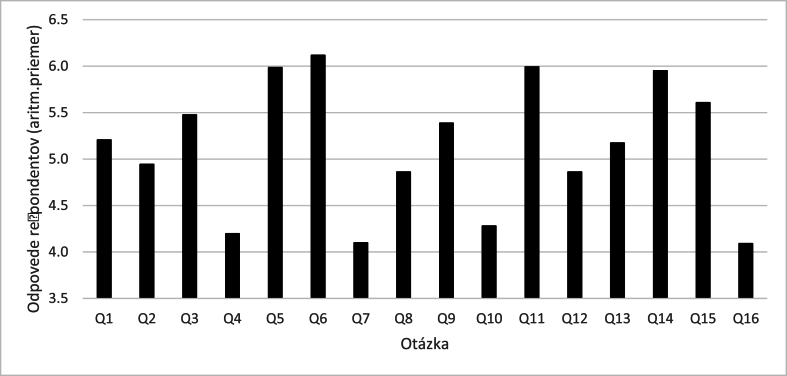
Average score of respondents' answers to questions Q1 - Q16.

When analyzing the research results, we first calculated the average score of the responses of all respondents for each questionnaire item (Q1-Q16) (Fig. 1).

Another method we used to analyze the research results is Factor Analysis (FA). The principle of FA is to explain a large number of measurable variables using a small number of variable-factors (latent variables), which are linear combinations of the original variables ([78,79]). Using factors, or latent variables that characterize the causes, and they are the source of variability, it is possible to reduce the number of variables while preserving the maximum information and to find a connection between the observed variables Q1-Q16 (observable or measurable causes) and new variables (factors). The result of solving factor analysis is a matrix of factor saturations. This matrix is a matrix of correlation coefficients between variables Q1-Q16 and latent variables, i.e., factors. High factor saturation means that the factor significantly affects the observed variable, or variable significantly affects the factor. A rule can be used for an approximate assessment of the significance of factor saturations: those factors whose absolute value is higher than 0.3 are usually considered statistically significant, and moderately significant factors with an absolute value greater than 0.4. Values of factor saturations whose absolute value is greater than 0.5 are considered very significant [79]. Before starting the analysis of the results of the experiment using FA, it is necessary to determine (extract) the number of latent variables - factors. We used the principal components method to determine the number of factors [80]. Using the method of principal components in the STATISTICA program, we obtained a table that contains eigenvalues, their percentage of total variability, cumulative eigenvalues and cumulative percentages (Table 1).

**Table 1:** Eigenvalues of the correlation matrix and percentage of explained variance.

Value number	Eigenvalue	% Total variance	Cumulative Eigenvalue	Cumulative %
1	5.110	31.935	5.110	31.935
2	3.040	18.997	8.149	50.932
3	1.710	10.686	9.859	61.618
4	0.976	6.099	10.835	67.717
5	0.753	4.708	11.588	72.426
6	0.636	3.974	12.224	76.400
7	0.618	3.863	12.842	80.263
8	0.542	3.385	13.384	83.648
9	0.519	3.241	13.902	86.889
10	0.428	2.674	14.330	89.563
11	0.400	2.498	14.730	92.060
12	0.341	2.129	15.070	94.190
13	0.300	1.874	15.370	96.064
14	0.249	1.554	15.619	97.617
15	0.218	1.360	15.836	98.977
16	0.164	1.023	16.000	100.000

According to Kaiser's criterion, the number of factors should be equal to the number of eigenvalues of the realization of the correlation matrix, which are greater than 1. Table 1 shows that this condition is met by 3 eigenvalues of the correlation matrix, which together (cumulatively) explain 61.6181 % of the total variance. Based on the mentioned criteria, we chose 3 factors for our case.

The FA also includes the correlation matrix of input variables Q1-Q16 (Table 2).In Table 2 we can see that (for example) the calculated value of the correlation coefficient between the answers to question Q3 and Q9 is 0.65. This means that there is a high degree of correlation between respondents' answers to questions Q3 and Q9. In other words, if the respondents chose low (or high) values from the offered options (scales) when answering question Q3 (The solved problem belongs to my area of interest), so also when answering question Q9 (I want to gain new knowledge from the given area) they chose low (or high) values. Conversely, if the answer values increase (or decrease) (respondents choose high answer values) for question Q9, then they choose high (or low) answer values for question Q3 as well (the correlation coefficient is symmetrical).

**Table 2:** Correlation matrix.

	Q1	Q2	Q3	Q4	Q5	Q6	Q7	Q8	Q9	Q10	Q11	Q12	Q13	Q14	Q15	Q16
Q1	1.00	0.23	**0.62**^a^	0.15	0.06	**0.33**^a^	0.20	0.24	**0.45**^a^	**0.34**^a^	0.03	0.27	0.11	0.29	−0.01	0.21
Q2		1.00	0.05	**0.46**^a^	0.24	0.24	**0.54**^a^	**0.57**^a^	0.12	**0.52**^a^	0.20	**0.59**^a^	**0.41**^a^	0.10	0.30	**0.61**^a^
Q3			1.00	0.01	−0.09	**0.39**^a^	−0.10	0.19	**0.65**^a^	0.17	−0.15	0.04	−0.01	**0.46**^a^	−0.13	0.02
Q4				1.00	0.23	0.13	**0.36**	**0.42**^a^	0.06	**0.48**^a^	0.12	**0.45**^a^	0.29	0.10	0.23	**0.52**^a^
Q5					1.00	−0.06	0.23	0.17	−0.07	0.14	**0.73**	0.13	**0.47**^a^	−0.11	**0.58**^a^	0.23
Q6						1.00	0.16	0.21	**0.35**^a^	0.08	−0.09	0.22	0.18	**0.51**^a^	−0.02	0.16
Q7							1.00	**0.46**^a^	0.00	**0.48**^a^	0.28	**0.54**^a^	0.29	−0.02	0.25	**0.68**^a^
Q8								1.00	0.26	**0.56**^a^	0.18	**0.53**^a^	**0.32**^a^	0.12	0.17	0.57
Q9									1.00	0.18	−0.09	0.13	−0.02	**0.61**^a^	−0.18	0.07
Q10										1.00	0.18	**0.52**^a^	**0.32**^a^	0.10	0.20	**0.64**^a^
Q11											1.00	0.17	**0.42**^a^	−0.18	**0.53**^a^	0.21
Q12												1.00	0.27	−0.01	0.23	**0.67**^a^
Q13													1.00	0.13	**0.38**^a^	0.37
Q14														1.00	0.01	0.07
Q15															1.00	0.27
Q16																1.00

a *p* < 0.05.

Analogously, based on the size of the calculated value of the correlation coefficient, we can also interpret the other calculated values of the correlation coefficients in the correlation matrix (Table 2).

**Fig. 2:**
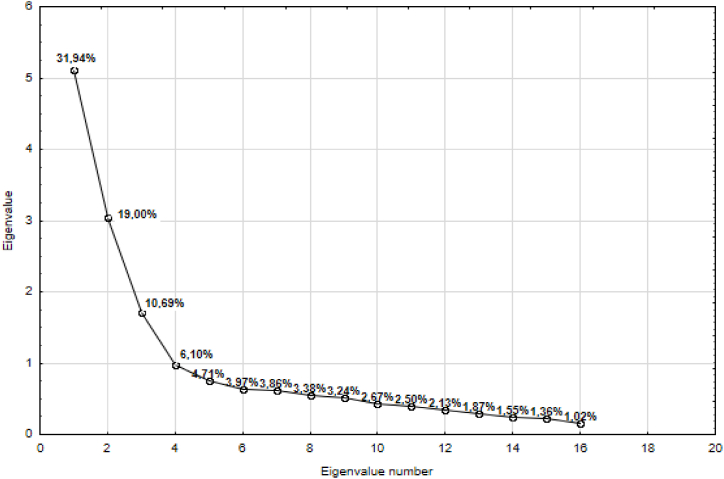
Correlation matrix eigenvalues and percentage of explained variance.

When performing FA for 3 factors, we reduced Table 1 for 16 variables for 3 variables and we can conclude that the first common factor explains 31.94 % of the variance contained in the 16 monitored variables, the second 19.00 % and the third factor 10.69 % of the variance. The total percentage of explained variance is 61.63 %. The situation is illustrated in Fig. 2.

The FA result has a rather complex structure because the first factor has higher correlations with all variables than the second factor because it explains the largest proportion of the variables' variability, etc. For a simpler interpretation of the results, it is advisable to go to the so-called a simple structure in which each factor is highly correlated with (and named after) several variables and its correlations with other variables are low. The transition to a simple structure is made possible by the rotation of the factor scheme – VARIMAX [81,82]. After the 1st rotation, we received the following estimate of the matrix of factor loadings (Table 3).

**Table 3:** Estimation of the matrix of factor loadings (saturations).

Variable	Factor 1	Factor 2	Factor 3
Q1	0.254	**0.665**^a^	0.045
Q2	**0.760**^a^	0.112	0.205
Q3	−0.006	**0.845**^a^	−0.084
Q4	**0.648**^a^	0.040	0.136
Q5	0.094	−0.042	**0.891**^a^
Q6	0.191	**0.626**^a^	−0.022
Q7	**0.747**^a^	−0.064	0.175
Q8	**0.724**^a^	0.208	0.105
Q9	0.076	**0.822**^a^	−0.099
Q10	**0.755**^a^	0.151	0.092
Q11	0.110	−0.107	**0.846**^a^
Q12	**0.805**^a^	0.056	0.064
Q13	0.337	0.116	**0.613**^a^
Q14	0.007	**0.768**^a^	−0.026
Q15	0.189	−0.086	**0.751**^a^
Q16	**0.864**^a^	0.020	0.148

a Loads greater than 0.45 are highlighted.

From Table 3, we see that the first factor is positively saturated with variables (questions) Q2, Q4, Q7, Q8, Q10, Q12 and Q16. Considering that questions Q2, Q4, Q7, Q8, Q10, Q12 and Q16 are mainly related to classroom climate, Factor 1 (F1) could therefore be named as **"Psychological Safety".**

**Fig. 3:**
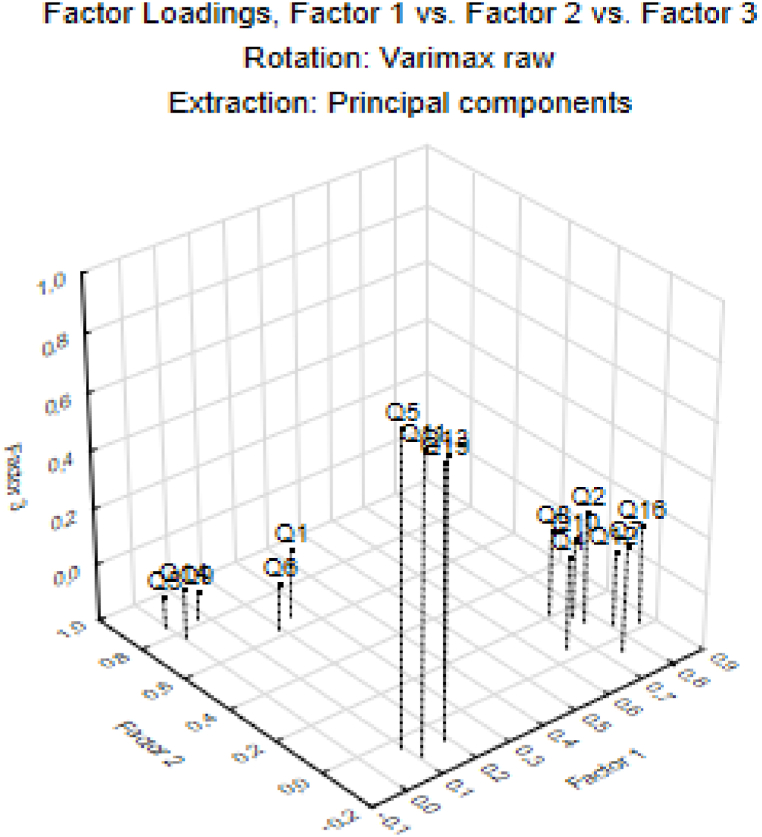
Representation of input variables in the factor space (3 factors).

The second factor is positively saturated with variables (questions) Q1, Q3, Q6, Q9 and Q14. Questions Q1, Q3, Q6, Q9 and Q14 of the used questionnaire refer mainly to the internal motivation of the respondent. For that we called Factor 2 (F2) "Personal Interest". The last, third Factor is positively saturated with variables (questions) Q5, Q11, Q13 and Q15. Since questions Q5, Q11, Q13 and Q15 mainly concern the respondent's willingness to cooperate in solving the problem, we called it **"Cooperation**“. Fig. 3 illustrates the saturation of calculated individual factors with input variables.

Based on the FA results, we found that the input variables Q1-Q16 form 3 varied factors, which we called: **Psychological Safety, Personal Interest** and **Cooperation**. We were interested in whether these differences between the three factors (dimensions) were also statistically significant. The tested problem is formulated as follows.

H0 The differences between Factors 1 to 3 are not statistically significant; against the alternative hypothesis

H1 The differences between Factors 1 to 3 are statistically significant.

Since the assumption of a normal distribution of the observed characteristics is not justified, we used the Friedman test to test the null hypothesis. The Friedman test is a generalization of the Wilcoxon one-sample test and is a nonparametric alternative to the bivariate analysis of variance with one observation in each subclass [83,84].

If we reject the tested hypothesis $H_{0\;}$ in favor of the alternative hypothesis $H_{1}$, the question remains unanswered, which selections are statistically significantly different from each other. We will use Neményi's method of multiple comparisons in the Friedman test to compare the differences between the individual selections in the Friedman test ([83,85]).

In our case, the observed characteristic X is the average values of the answers to the questions in the three established factors F1, F2 and F3. We observe the quantity X on 244 units (students) of the sample set in *k = 3* factors. We will verify the validity of the null hypothesis $H_{0\;}$ that all selections come from one distribution using the Friedman test using the STATISTICA program. In our case, we calculated the test criterion value Q=285.946 and the probability value p=0.000.

The output of the computer also includes a table (Table 4) in which the average ranks, sums of ranks, arithmetic averages and standard deviations of the values of the observed character in individual thematic units are listed.

**Table 4:** Results of the Friedman test.

Variable	Average Rank	Sum of Ranks	Mean	Std.Dev.
F1	1.123	274.000	4.475	0.663
F2	2.365	577.000	5.593	0.671
F3	2.512	613.000	5.689	0.722

Since the calculated probability value p < 0.01, we reject the null hypothesis $H_{0\;}$ at the significance level α = 0.01. This means that the observed differences between the average values of answers to questions in individual factors are statistically significant.

Next, we were interested in which of the factors F1, F2, F3 are different with respect to the average values of the answers to questions Q1-Q16. When comparing the differences between individual sets in the Friedman test, we will use the Neményi method: we will compare the absolute values of the differences of the order sums in the individual levels of the time factor with the critical value, which we will calculate according to the relation

$$q_{k,\infty }\left(\alpha \right)\sqrt{\frac{1}{12}nk\left(k+1\right)}=62.48\text{.}$$

The absolute values of the differences of the order sums in individual levels of the time factor are clearly written in the following table (Table 5).

**Table 5:** Absolute values of the differences of the rank sums in individual factors.

ǀF1- F2ǀ	303.00^a^
ǀF1 – F3ǀ	339.00^a^
ǀF2 – F3ǀ	36.00

a Values exceeding the critical value.

If we compare the values shown in Table 5 with the critical value 62.48, we can conclude that there are statistically significant differences in two cases.

1. between factors F1: "Psychological safety" and F2: "Personal interest".
2. between factors F1: "Psychological safety" and F3: "Cooperation".

**Fig. 4:**
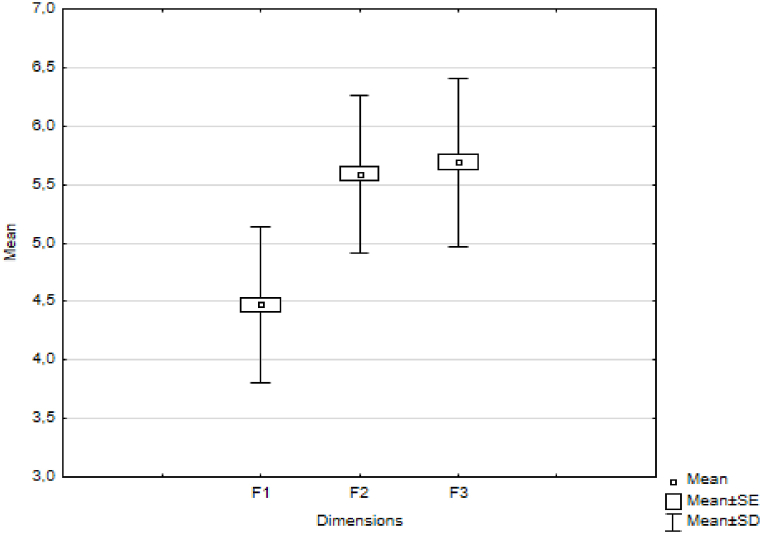
Average scores for individual factors (dimensions).

The situation is illustrated in Fig. 4.

## 5 Findings from interviews

The statements of the interview participants confirmed the finding from the quantitative part of the research that the students' decision to participate in the joint solution of the task is positively influenced primarily by the interest in acquiring new knowledge. Among the students, this interest was conditioned by several factors, among which a positive attitude towards the given subject prevailed. An interesting finding is that the majority of students expressed their desire to acquire such a volume of knowledge that it would be enough for them to "pass" to the next year. Thus, the identified main motivational factor (Personal interest) in favor of engaging in joint task solving is part of the internal motivation to fulfill one's own study goals. S4 expressed himself most clearly in this regard, saying:

"I will participate in the joint solution if the correct solution proposals are awarded with bonus points that will help us in the overall evaluation of the subject."

An interesting finding is the students' belief that only those who understand the problem being solved can participate in joint work. Almost every interviewee stated that he would not get involved in solving a task if he did not have sufficient knowledge that would give him a feeling of certainty that his proposed solution or answer to the question would be correct. For example, S2 commented as follows:

"If it seems to me that a classmate, who is better at mathematics than me, has made a mistake, I prefer not to say anything and change the solution of the problem according to his solution procedure. He's smarter than me, so his solution is definitely better than mine."

The need for a sense of team psychological safety and trust in the collective was evidenced by the statements of all the students who participated in the interview. Almost every one of them mentioned the fear of being ridiculed, primarily by the teacher and only secondarily by classmates. Only S7 gave the seemingly opposite order:

"For me, the reaction of my classmates is probably more important, because the teacher has experience with bad answers and doesn't really show his negative reaction."

The fear of ridicule also resonated in statements about the need for sufficient knowledge, as a necessary condition for participation in joint activities. However, S1 expressed that the effort to gain new knowledge gives him the motivation to overcome the fear of ridicule. S1 said:

"If I don't understand something, I'd rather log in and have it explained. I'd rather be laughed at than not have it explained to me.''

The interviews with the students generally revealed the important role of the teacher in deciding to participate in a joint activity in the classroom. Students reported that positive feedback and encouragement from the teacher, even when they make a mistake, gives them the courage to re-engage in the activity. However, they considered more important the way the lesson was conducted, a positive relationship with the teacher and the ability of the teacher to explain the subject in different ways. S3 made a unique statement in this regard:

"If the teacher is like google translator and tells me one thing in multiple ways, it's impossible NOT to engage."

Students are also aware of the need for cooperation in achieving their own goals. Several stated that they engage in solving tasks primarily for the purpose of improving their knowledge. At the same time, they primarily expect cooperation from the teacher, because for them it is the teacher who guarantees the correctness of the procedure. They are more reserved about suggestions for solutions to tasks from their classmates, because the number of imprecise or incorrect suggestions creates confusion for them. S5 made an interesting comment:

"Sometimes I prefer not to listen to the suggestions of my classmates and wait for the teacher to propose. It's because at the end of the discussion I don't really remember what anyone said, I'm in a mess and I often don't know how to properly proceed with the solution."

## 6 Discussion

Based on the results we obtained using factor analysis, we found that the items of the questionnaire form three varied factors that need to be analyzed separately. In the F1 factor, the average score of the respondents' answers was 4.475, which corresponds to a moderately strong influence of the "Psychological safety" factor on the motivation of students to engage in joint activities during classes. Within the F1 factor, variables Q2, Q8 and Q12 (rather strong influence) achieved the highest scores, which represent the teacher's and classmates' reactions to a possible mistake made by a student when presenting his own proposal for solving a problem. Our findings indicate a strong link between a sense of team safety and a positive error climate in the classroom. This leads us to the conclusion that the teacher's attitude towards errors is not only a decisive factor in the creation of a positive error climate in the classroom, as claimed by previous research (e.g. Refs. [[13], [14], [15]]), but also a primary element in building a sense of team psychological safety within classroom learning.

On the other hand, variables Q4, Q7 and Q16, regarding relationships between classmates, were perceived by the respondents as elements with a moderately strong positive influence on their decision to engage in joint activities within teaching. The analysis of the "Psychological safety" factor shows that the willingness of students to engage in teaching activities is more supported by minimizing the risk of negative reactions from classmates than by mutual relationships in the classroom. This conclusion points to the importance of adaptive error management by the teacher. Adaptive error management involves discussing with the whole class the significance of one's own errors in learning, thereby preventing negative reactions from classmates ([19,86]). Several studies (e.g. Refs. [37,[87], [88], [89]]) show that adaptive error management creates a positive error atmosphere in the classroom. Based on our research, we can supplement this knowledge with the fact that adaptive error management has the potential to build a sense of psychological safety in the classroom. In accordance with the conclusions of Appelbaum et al. [65], also in our research, from a more detailed analysis of the "Psychological safety" factor, it follows that the teacher creates an atmosphere of trust within the framework of learning through adaptive error management, which is manifested in the individual by an increased feeling of team psychological safety, which subsequently leads increased willingness to participate in common activities in the classroom.

The average score of the factor F2 ("Personal interest") is 5.593, which we could interpret so that the factor has a strong positive influence on students' decisions about whether they are interested and willing to engage in active learning. Of the variables that saturate this factor, variables Q6 and Q14 achieved the highest scores, which we can interpret as the student's internal interest in solving the assigned task. This interest, which is stimulated by internal motivation, leads the student to the decision to present his proposals for solving tasks in front of the collective. Personal interest in solving a given problem or acquiring necessary new knowledge has the potential to encourage active participation in the learning process in the classroom. Due to the fact that variable Q6 (I wonder if I am thinking in the right direction.) achieved the highest score of all variables, we conclude that the internal interest in solving the task is connected with the need to correct possible errors. We came to the same conclusions as Covington [90], according to which internal motivation can be described with the words "I enjoy it" or "I am interested in it", and in this case the student is willing to take the risk represented by the presentation of his own opinions and solutions in front of the class group. In addition, the interviews revealed that the effort to solve the task can be motivated by the personal benefit of the student, whose goal is to successfully complete the subject.

We calculated the highest average score of 5.689 in the case of factor F3 "Cooperation", which means that the factor "Cooperation" strongly positively affects the willingness of students to present their opinions when solving problems together in the classroom. In factor F3 "Collaboration", variables Q5 and Q11 achieved the highest scores, which indicate the need for trust in the collective in which learning takes place. According to Robinson [91], trust is the expectation that the actions of others will be favorable to one's interests, so that one is willing to take risks for these actions. In this context, we can interpret our finding as a strong need for mutual trust between classmates when deciding to risk a possible error when presenting their own proposals for solving a problem. At the same time, these findings point to the willingness of an individual who is internally motivated to learn new knowledge, to use the help of classmates, and thereby increase the effectiveness of learning in a team. According to several studies (e.g. Refs. [92,93]), classmates may be more aware than their teachers of what other students do not understand and will often provide explanations that better clarify a classmate's misconceptions. At the same time, mutual cooperation builds intuition for each other's needs [94]. However, the interviews indicated that students are not fully aware of the benefits of mutual cooperation and often try to cooperate more with the teacher. It is probably conditioned by the desire to have correct solutions without unnecessary "wanderings", which can cause the student not to know the correct solution procedure after solving the task [26]. Based on our research, we conclude that mutual cooperation between students will be more intense if mutual trust is created between them.

The results of the statistical analysis confirmed statistically significant differences between the average value of the answers in the factor F1 ($\bar{x}=4.475$), compared to the factors F2 ($\bar{x}=5.593$) and F3 ($\bar{x}=5.689$). Based on the above results, we can conclude that when a student decides whether to participate in joint activities and thus reveals a possible mistake he has committed, the student is statistically significantly more motivated to participate in activities by the factors "Personal interest" and "Cooperation" than by the "Psychological safety" factor. Based on our research, we can also conclude that the feeling of psychological safety has a strong motivational potential when the student decides to reveal his own mistake. The students' answers reveal a close connection between the feeling of psychological safety and the absence of negative reactions to their possible erroneous statements. Therefore, we come to a similar conclusion as reached by Pan et al. [42] and that building a sense of psychological safety in the classroom, on which the teacher has a decisive influence, can eliminate students' aversion to making mistakes, which significantly reduces students' engagement in active learning. A similar conclusion was reached by Refs. [13,14].

In further research, it would be necessary to examine the interrelationship of the dimension (factor) "Psychological safety" with the remaining two identified factors. According to our findings, the "Personal interest" dimension, which can be considered as a manifestation of internal motivation to acquire new knowledge, has a stronger motivational potential when a student decides to engage in active learning. Due to the motivation to engage in active learning, students perceive this dimension as equal to the "Cooperation" dimension. According to Kuhn [95] cooperation is one of the tools for obtaining explanations that the student needs during learning. However, according to Gillies [96], being a member of a group is not enough to create cooperation between classmates. Based on the results we obtained in our research, we conclude that a key factor supporting the creation of cooperation in a team is mutual trust, which is connected to the feeling of team psychological safety. However, this claim would need to be verified by further research.

Based on our research, we conclude that the feeling of psychological safety also plays a key role in the school environment. We believe that if the teacher succeeds in creating this feeling in the students during the teaching process, it will significantly increase the students' involvement in joint activities and create a suitable environment enabling effective learning from mistakes. However, the feeling of psychological security itself may not be the strongest motivating factor. According to our findings, the student's internal interest in gaining new knowledge is a stronger motivator, as a result of which he is more willing to enter into cooperation, which can take the form of explanations, giving instructions, pointing out mistakes or handing over materials necessary for the given task, which corresponds to the results they reached [97]. According to Gillies and Ashman, cooperation motivated by the desire for new knowledge develops in students an intuitive sense of the needs of others and they learn to provide help to others when they feel it is necessary ([94,98]).

## 7 Study limitations

Some limitations of our study may have affected our results. Students from secondary schools, where various forms of active learning are used, participated in the pedagogical experiment. The same forms of active learning can be implemented in different ways, which can have an impact on the degree of student involvement in joint activities. This factor could affect the results of our research, because the students' statements in the questionnaire and in the interview are also conditioned by what active learning practices the students experienced during their studies. In further research, it would be appropriate to check what forms of active learning support the building of a sense of team psychological safety and thereby increase the willingness of students to engage in joint activities.

## 8 Conclusion

Current trends in education place the student at the center of education and the teacher should take on the role of facilitator. Active cooperation is expected from the student in the education center, which is associated with fears of expressing their opinions, because a possible mistake may result in a negative reaction from the environment. According to our findings, a very strong motivator to participate in joint activities is the student's internal motivation to acquire new knowledge. A student motivated in this way is also ready to cooperate with classmates. Our research pointed to the important role of the teacher in managing joint activities in the classroom. With its adaptive management, it not only creates a positive error climate in the classroom, but also builds a sense of psychological security for the student. This feeling gradually turns into trust in the class team and the student willingly participates in joint activities. We think that the teacher, with his positive attitude towards mistakes, can significantly support the student to become the center of education. The cooperating student can then benefit from the benefits of active learning during their learning.

## Ethical approval

Ethical approval was obtained from the Ethic Committee of the Constantine the Philosopher University in Nitra under the number UKF/894/2024/191013:002 (chairman Prof. Dr. M. Bauerová). The Ethics Committee stated and confirmed that the research does not contradict any ethical rules and confirms that all respondents were informed about the use of their answers. The questionnaire was filled out anonymously. All respondents were initially informed about the objectives of the research and the use of the questionnaire. By filling out the questionnaire, they agreed to its evaluation.

## Data availability statement

Data will be made available on request.

## CRediT authorship contribution statement

**Dalibor Gonda:** Resources, Investigation. **Anna Tirpáková:** Visualization, Data curation. **Gabriela Pavlovičová:** Validation, Methodology. **Viliam Ďuriš:** Formal analysis, Conceptualization.

## Declaration of competing interest

The authors declare that they have no known competing financial interests or personal relationships that could have appeared to influence the work reported in this paper.
