# Supplementary material for: The role of a team psychological safety feeling in teamwork in the classroom
Source: Heliyon. 2024 Sep 7;10(18):e37618. doi: 10.1016/j.heliyon.2024.e37618 (PMC11415665; doi:10.1016/j.heliyon.2024.e37618)
Supplement: Multimedia component 1 [file mmc1.docx]

Appendix A

Participating in solving a common problem within teaching motivates me:

1. My classmates do not blame me for the mistake I make.

2. The teacher does not evaluate me based on the mistakes I made while learning.

3. The solved problem belongs to my area of ​​interest.

4. My classmates do not reject someone if they are different.

5. I assume that my solution can inspire others.

6. I wonder if I am thinking in the right direction.

7. Risks are worth taking in my class.

8. The teacher's facial expression and reactions are the same for correct and incorrect answers.

9. I want to gain new knowledge from the given field.

10. I want to help solve the problem.

11. It is not difficult for me to ask a classmate for help.

12. No one disparages my answers or suggestions for solving a problem.

13. Working with my classmates, I feel that my unique skills are appreciated and used.

14. I wanted to solve an interesting problem, but I needed help.

15. Every idea can be useful.

16. In my class it is possible to talk about potential problems and difficulties.
